# Vaginal microbiomes and their pertinent social context: a microbial ecological review proffering AMR-STI acquisition and emergence

**DOI:** 10.1186/s13293-026-00953-2

**Published:** 2026-07-14

**Authors:** Samantha J. Webster, Ian E. Cock, Carney Matheson, Emma L. Sweeney

**Affiliations:** 1 School of Environment and Science, Griffith University, 170 Kessels Rd, QLD 4111 Nathan, Australia; 2 Institute for Biomedicine and Glycomics, Griffith University, 1 Parklands Drive, QLD 4215 Southport, Australia; 3 The Frazer Institute, Royal Brisbane & Women’s Hospital Campus, The University of Queensland, Building 71/918, QLD 4029 Herston, Australia

**Keywords:** Sexually transmitted infections, Antimicrobial resistance, Vaginal microbiome, Microbial ecology.

## Abstract

**Background:**

In the landscape of sexual health, sex, gender, and sexuality are inextricably linked and highly relevant to sexually transmitted infections (STIs). Globally, key sexual and reproductive health concerns of women have been associated with the socioeconomic status of their country, indicating that social context bears influence over sexual health outcomes. Further, the increasing prevalence of antimicrobial resistant STIs (AMR-STIs) in the sexual networks of gay and bisexual men-who-have-sex-with-men (GBMSM) suggests an implicit connection between microbiological and social phenomena, although research to date is relatively limited and often fails to reflect the complexity and nuance of sexual networks. Vulval and vaginal microbiome composition may influence STI acquisition and transmission, yet the relationships between composition, microenvironment, and STIs remain largely overlooked, especially in the context of women and gender-diverse people. In this article, we explore the possibility that a combination of social, sexual, and behavioural factors, combined with biological features, shape the microbiological context of STIs within the vaginal microenvironment.

**Main:**

The human vaginal microbiome (VMB) forms an ecological niche home to a complex ecosystem of microorganisms. The microbial composition of the VMB is diverse between individuals, with variations observed across racial and ethnic groups, and intrapersonal fluctuations linked to a plethora of factors both within and outside of personal control. Importantly, VMB health is a crucial component of wellbeing for people assigned female at birth (AFAB), transgendered women with neovaginas, and their sexual partners. Clinical context also remains important; in Australia, doxycycline prophylaxis (Doxy-PEP) has recently become available to GBMSM networks aimed to protect against the acquisition of STIs. However, Doxy-PEP guidelines exclude AFAB people and fail to specify regarding use among gender diverse individuals. Given the high prevalence of AMR-STIs within GBMSM networks, the impact of this intervention on excluded partners should be thoroughly investigated. Factors in the VMB such as biofilm formation and necessary microbial balance with opportunistic pathogens renders this ecological microbial niche a hypothetically perfect platform for AMR development and emergence within the social context.

**Conclusion:**

This review explores the social context of vaginal microbiomes, their potential influence on AMR-STI development, and highlight several important knowledge gaps to benefit from further research.

## Plain language summary

Sexual health is shaped by both social factors, such as sex, gender, and sexuality, and biological factors, including anatomy, microbial ecosystem present in genitalia, and exposure to sexually transmitted infections (STIs). Globally, STIs are becoming increasingly drug-resistant, partially due to antibiotic overuse, producing ‘AMR-STIs’ (STIs with antimicrobial resistance properties). Because STIs are highly prevalent in gay and bisexual men, and vaginal infections with STIs are less likely to display symptoms, women and gender diverse people have been largely overlooked as STI reservoirs. This is worrying given that treatments are provided based on available research, raising concerns about their effects on those key groups without sufficient research to confidently inform treatment. For instance, in Australia, Doxy-PEP (proactively using an antibiotic to prevent STIs) is available to gay bisexual and other men who have sex with men, but excludes people with vaginas and fails to address gender diverse individuals.

This review explores how social and biological factors interact with the vaginal microbiome (the community of microbes in the vagina) to influence the spread of both STIs and the genetic material which leads to AMR-STIs. We discuss the context of multiple types of vaginal microenvironment and highlight how race, menstrual milestones, and lifestyle factors may shape the balance of the vaginal microbiome, influencing STI risk. We argue that these intersecting biological and social factors likely play a key role in how STIs develop and spread. Hence, this review calls for more research into how these factors shape STI risk in the vaginal microbiome, how these factors shape risk for sexuality and gender diverse individuals, and ultimately how these factors influence the rise of AMR-STIs.

## Highlights

Vaginal microbiome (VMB) composition influences sexual health and forms a crucial component of wellbeing for people with vaginal physiology and their sexual partners.

Intersecting social, sexual, behavioural and biological factors shape the microbiological context of sexually transmitted infections (STIs) and can select for antimicrobial resistance (AMR-STIs)

Antimicrobial treatments applied to specific population groups can act as selective pressure for AMR-STIs and require significant pragmatic study with consideration to downstream effects.

Systematic historical neglect has led to gaps in understanding and medical treatment options for women and sexuality and gender diverse minorities seeking sexual and reproductive health care

Vaginal microenvironments form a key ecological niche for AMR-STI development and emergence due to differential clinical treatment, vaginal physiology, VMB ecology, social contexts, and intersecting knowledge gaps.

## Background

Sexual health is a critical, -yet typically overlooked- component of wellbeing [1–3]. Contemporary literature is increasingly recognising sexual identity as an integral component of an individual’s life, although the centrality of this varies across disciplines. Whilst sexual health is multi-faceted, sexually transmitted infections (STIs) represent a universal threat, and understanding their driving biosocial mechanisms is highly important. To that end, investigating biological nuance and confounding social complexities together may yield significant advances in our understanding of STI evolution, transmission and emergence. A key focus of this review is the microbial ecology -termed ‘micro-ecology’ throughout- or interactive microbiological ecosystem involved in the pathogenesis and lifecycle of bacterial STIs.

Part of the complexity of STI research lies in the interaction between sex, gender, and sexuality which together shape the sexual network dynamics underpinning transmission, which remain poorly understood. Sexual health research involving sexuality and gender diverse minorities (SGM) has traditionally treated LGBTQIA+ people as a uniform community [4] or directly compared them to ‘cishet’ -cisgendered heterosexual- reference populations as opposed to truly representative comparison cohorts. This approach obscures our understanding of what might constitute a ‘healthy’ microbial baseline for specific SGM subgroups [5, 6]. Some subgroups, such as gay or bisexual men-who-have-sex-with-men (GBMSM), have received a considerable amount of research attention, yet the literature continually lacks a clear characterisation of the ‘healthy’ microbial profile specific to GBMSM. This gap is important because differences in sexual practices and network structures from their cishet counterparts could contribute to differences in micro-ecological composition [7]. It follows that when these differences are not adequately accounted for, abstract biological phenomena may be interpreted relative to cishet baselines, increasing the risk that normal variations within SGM populations is unfairly pathologised.

In the context of antimicrobial-resistant sexually transmitted infections (AMR-STIs), clinical research tends to be largely reactionary and somewhat GBMSM-centric, yet a clear understanding of the evolution, emergence, and transmission of AMR-STIs often remains elusive [8, 9]. This is part of a complex and nuanced set of data challenges encountered when considering the intersecting biological and social drivers of STIs. Firstly, there are -and have historically been- valid epidemiological reasons to prioritise STI research for GBMSM, including their disproportionate burden during the HIV/AIDS (Human Immunodeficiency Virus/Acquired Immunodeficiency Syndrome) epidemic [10–12]. However, a legacy of such targeted approaches can also shape how STI epidemiology is observed and interpreted. In particular, differential STI testing intensity across populations can also introduce surveillance bias [13, 14]. Frequent and reflex testing strategies are important methods of improving detection for groups at increased risk of exposure such as GBMSM, and also increase the likelihood of detecting asymptomatic infections. Because many STIs are asymptomatic, GBMSM demonstrate a high rate of sexual health literacy and undergo STI testing more frequently than their cishet counterparts, this can lead to higher case ascertainment for GBMSM [14, 15]. Consequently, surveillance data may over-represent populations like GBMSM which are subject to more intensive testing, and functionally obscure the true distribution of infection across the population, or the intensity of the true distribution.

Secondly, targeted testing and research frameworks which directly compare GBMSM and cishet populations risk oversimplifying STI epidemiology by focusing on identity categorisation as opposed to sexual network dynamics. Infection risk is mediated by complex network connectivity patterns and biological pathogenesis [1]. Hence, identity driven approaches may obscure important heterogeneity within and across populations and fail to adequately represent the experiences of diverse SGM. Simply put, this approach doesn’t reflect the reality that sexual networks are highly complex, and fails to adequately capture SGM, creating downstream impacts borne disproportionately by SGM. Intersectional frameworks are key in identifying areas to increase research efforts. For instance, cis-male sexual health benefits from STI investigations into both heterosexual and GBMSM networks; however, subgroups within the LGBTQIA+ community are inadequately captured by heterosexual-focussed research and do not attain similar benefits from this research [6, 16–19]. This important gap provides a clear opportunity for researchers; sexual network dynamics require demystifying, and other SGM would directly benefit from additional research focus. Moreover, a focus on improving sexual healthcare for SGM is timely; stigma has played a considerable role in preventing healthcare access for SGM, although this does appear to be lifting. In Australia, the National Census is set to include gender identity and sexual orientation questions for the first time in 2026 [20], and the Royal Melbourne Hospital recently implemented its own SGM data collection program [21]. This is promising as the collection and investigation of social metadata in connection with biological phenomena is clearly necessary to elucidate the mechanisms that underpin both sexual health and sexually transmitted infection (STI) dynamics.

Finally, we must consider how AFAB people fit into this context. Women’s sexual health has long been systematically overlooked, and is often reduced to obstetric and reproductive focuses, with female genital anatomy traditionally ignored outside of its reproductive or aesthetic function [22]. Notably, surveillance bias is also relevant when examining STI trends of cishet women, which are undergo STI testing more than their male counterparts [23, 24]. Mystifying trends further, AFAB SGM are poorly recorded in health surveillance and research, though modern studies are beginning to explore differences in VMB compositions between relevant subgroups [see Sect. "The Vulval and Vaginal Microbiomes"]. Exacerbating issues, the consistent underfunding of research in this area has led to a degree of ignorance, which is further compounded for women of colour, gender-diverse people, and individuals suffering multiple degrees of marginalisation [25]. Vaginal health is increasingly understood to be driven by microbial interactions and composition within the vaginal microenvironment [26, 27]. However, the microbial ecology of STIs in this context is poorly understood. Biological research endeavours frequently overlook the social contexts shaping STI emergence, focussing on the ‘who’ and ‘what’ of incidence and prevalence over the mechanistic ‘how’. Hence our current understanding of STIs and the mechanisms driving their emergence and evolution are limited by our poor understanding of both the microenvironments they inhabit, and the relevant social contexts which influence them. This functions as a self-limiting mechanism for the development of meaningful preventative strategies and medical interventions, which is deeply concerning in the era of AMR-STIs where treatment options are becoming increasingly scarce. There are fewer antimicrobials progressing through commercial development, and STIs are now expanding beyond their previous demographics. As outlined in the model of sexual wellbeing proposed by Mitchell et al. [28], sexual health and social contextual factors are difficult to extricate, with each of these contributing to the overall shape of the microbiological context.

**Fig. 1:**
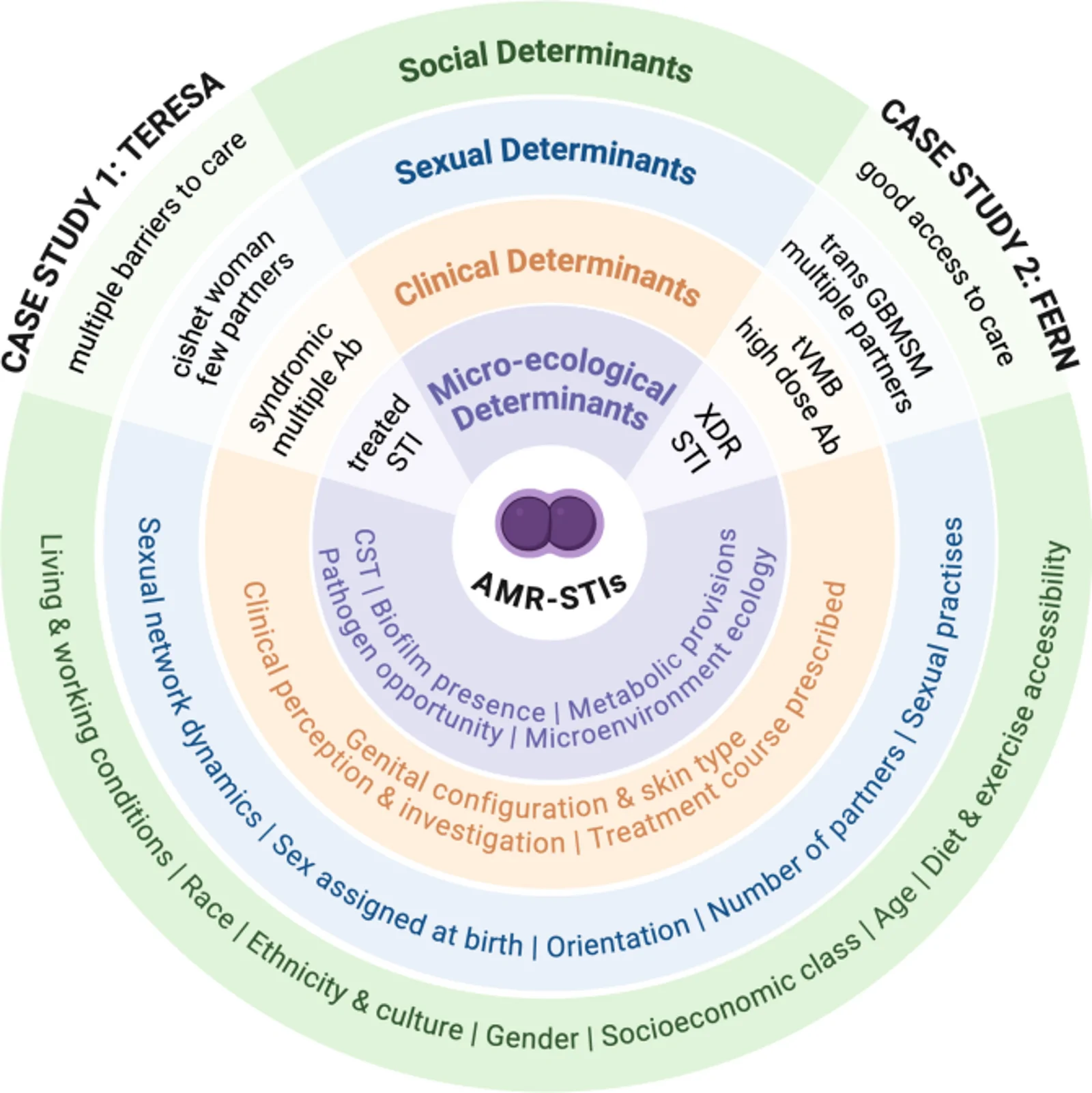
**A** Conceptual framework exploring multiple determinants of sexual health and their influence on STIs. Our framework maps the intersection of factors which facilitate the emergence of AMR-STIs. We advocate that the broader rings of influence such as social determinants of health (green), individual level sexual determinants (blue), clinical determinants (orange), and micro-ecological determinants (purple) each intersect to shape and influence AMR-STI emergence. For instance, the epithelial cell type in the vaginal lining or hormones in the vaginal microenvironment bear strong influence over VMB micro-ecology but also determine clinical treatment and interpretation of symptoms. Sexual determinants such as sex, gender, sexuality and sexual behaviours are influential for AMR-STI emergence and transmission but are also influenced by social context. Each of these factors may in turn influence clinical care (orange). Altogether, these concentric factors enable the emergence and evolution of AMR-STIs. Note that detailed explanation of case studies referenced in this figure are presented in Box 1 below. Fictitious case studies were constructed from real world scenarios [29–36]. STI - sexually transmitted infections; CST - community state type; tVMB – testosterone dominant vaginal microbiome. Created in BioRender [37]

This review discusses sexual and reproductive health (SRH), confounding factors which influence microbial ecology, and subsequent emergence and persistence of AMR- STIs. Specifically, we explore how the microbial ecology of the vaginal environment is influenced by (i) social contextual factors, (ii) health determinants, which may or may not be controlled by the individual, (iii) microbial factors, and lastly, we discuss the interplay of these factors with (iv) AMR-STIs. Given the scale and complexity of the issue, we propose a framework which integrates social, sexual, and microbiological determinants of sexual health through the lens of microbial ecology [Figure 1]. By applying this framework to both research and public health endeavours, the mechanistic and currently elusive driving factors of STI evolution may be better investigated.

Box 1: Structural and microbial determinants in sexual health - two contrasting cases To illustrate how our conceptual framework functions, we have constructed two fictitious case studies grounded in relevant epidemiological information [29–36].

**Case study 1: Teresa.** Teresa is a 23-year-old Aboriginal cishet woman living in remote Queensland. After weeks of intermittent symptoms, she takes time off work to drive to the nearest clinic and presents with pelvic pain and abnormal discharge. She undergoes STI testing and is treated at the appointment to avoid potential loss to follow up. Teresa’s syndromic case is managed as cervicitis with risk of PID, and she receives a single injection of ceftriaxone, a 7-day oral course of doxycycline, and a 7-day oral course of metronidazole. Her culture swabs are delayed in transit and do not yield gonococcal growth once they reach the lab several days later, however the diagnostic PCR tests positive for chlamydia and gonorrhoea. When she returns several months later for an unrelated condition, her clinician learns that her symptoms resolved following treatment.

**Case study 2: Fern.** Fern is a 31-year-old transgender man (GBMSM) living in metropolitan Queensland who uses HIV PrEP and demonstrates a high level of sexual health literacy. He is on long term testosterone therapy and undergoes regular STI screening given his casual engagement with GBMSM networks. Because he has a vagina, he is not recommended doxyPEP, though it is popular in his sexual network, and consequently Fern regularly uses condoms but is also having condomless sex with some partners more often. He has been struggling with vaginal dysbiosis symptoms of late and is unsure about estradiol gels, so initially dismisses abnormal discharge. Following asymptomatic testing, Fern tests positive for gonorrhoea and is immediately treated with a single injection of ceftriaxone and oral azithromycin. At his follow up test of cure 2 weeks later, culture swabs are taken which yield highly resistant gonococcal isolates, and Fern’s diagnostic PCR continues to test positive. Public health mechanisms are engaged which include contact tracing and whole genome sequencing, and Fern’s treatment is managed by public health microbiologists and infectious disease consultants. This likely involves a hospitalisation with high-dose intravenous antibiotics as well as testing and treatment of known sexual contacts.

**Reflections gleaned from applying the framework:** These fictitious cases present real biosocial issues experienced by patients and illustrate contrasting and complementary pathways to STI treatment. Teresa’s case involved structural barriers to care, syndromic presentation and an empiric approach with minimal microbiological confirmation to avoid loss to follow-up. In contrast, Fern’s case highlighted the limits of an empiric approach in an era of AMR-STIs and illuminated some finer points of this biosocial problem which required microbiological guidance to resolve. Comparing these cases highlights a distinct concern to emerge; what if a third unexamined case connects both Teresa and Fern’s sexual networks, presenting with multiple barriers to care? Together, these cases draw attention to existing tensions in sexual health systems. Epidemiologically driven treatment is essential, especially where barriers to access prevent microbiological insight, which is in turn critical for managing and monitoring resistance. Most importantly, neither approach is sufficient alone, with public health requiring integration of these strategies to manage evolving AMR-STIs.

## Sex matters: how sexual determinants shape the vulvovaginal microenvironment

Vaginal microbiome (VMB) composition is influenced by a dynamic interplay between host microenvironmental factors and several sexual determinants. Thus understanding how the VMB composition is shaped requires moving beyond purely microbial frameworks to consider how social context, including gender norms and sexual partnership patterns, shapes exposure, susceptibility, and micro-ecology. In this section we discuss the roles of the more socially-driven sexual determinants including gender and sexual network dynamics before addressing micro-environmental factors separately (see Sect. "The Vulval and Vaginal Microbiomes").

### Gender determines key issues and clinical care

The interplay between gender norms and SRH outcomes are complex. Factors including social stigma, poor education, limited autonomy, and entrenched power imbalances consistently result in poorer outcomes for individuals [1]. Sociocultural factors shape access to SRH care globally, yet distinctions can be made according to socio-economic context. Restricted autonomy and religious or cultural values were found to drive poorer SRH outcomes in low- and middle-income countries, whilst provider bias, cultural stigma, and socioeconomic disparities appear to form persistent barriers in high-income countries [1]. Essentially, this means that whilst barriers to SRH care may differ per social context, they do persist for at least some social groups in all settings. This holds true in Australia, where despite a high standard of healthcare, geographical disparity forms a key barrier to access for SRH care. A recent Australian study [38] identified that women living outside of major cities face limited agency in accessing SRH care due to poor service availability, prohibitive costs, diminished health literacy, and negative provider attitudes, which further exacerbate health inequities for rural communities. In this way, social factors play a role in determining SRH care, and hence arguably act as a driver of biological phenomena.

One pertinent example of social mechanisms driving STI emergence comes from syphilis trends in Australia. A syphilis epidemic was declared in rural Northern Queensland in 2011. Initially the outbreak disproportionately impacted First Nations Peoples and GBMSM, yet has now expanded alarmingly in South-East Queensland and in neighbouring jurisdictions as well as into the broader population, including women of reproductive age [39–41] and > 100 cases of congenital syphilis across Australia, leading to the recent declaration of syphilis as a Communicable Disease Incident of National Significance [42]. Tension has increased within the rural communities initially affected by the outbreak; particularly as national support efforts have seemingly expanded alongside the resurgence of syphilis in metropolitan areas. In the context of Northern Queensland, where there is a higher proportion of First Nations Peoples, questions around healthcare access and inequities have been raised and warrant careful consideration. More broadly, it is clear that the patterns of syphilis emergence, transmission, and detection are shaped not only by biological factors, but also by social determinants and governmental policy.

This example provides a clear picture of two key principles; (i) how social issues such as access to healthcare, education, and social stigma, can drive biological phenomena in specific demographics, and (ii) how STI transmission dynamics do not obey traditional social constructs, nor the tidy divisions of public health categories. Relevant to the latter point, reductive divisions often fail to account for the complex nuance of sexual behaviour [43]. This is important given our healthcare guidelines often rely upon either accepted social constructs or clear data, yet sexual transmission involves a complex intersection of sex, gender, and sexuality, which is not well recorded. So, what happens when the situation is more complex and -as in the case of STIs- nuance is required? It makes sense to consider STIs in the context of how SRH care and gender intersect at the clinical level to influence biological phenomena.

Knowledge of female anatomy and physiology remains poor, even among healthcare providers [44]. Medical research has historically excluded female participants for paradoxical fears of the female reproductive system [either negatively impacting results or conversely being adversely affected by study treatments]. This systematic exclusion of women from medical trials has meant that the findings of male clinical trials were applied to women as a general rule [45–54]. We now know this is not reflective of reality. A surplus of evidence states that failing to research female patients fails women and leads to detrimental health outcomes [see [55]**]**. Patients who are LGBTQIA+ likely to experience poorer SRH compared to their heterosexual and cisgender counterparts [56, 57]. Applying an intersectional lens to this issue yields similarly distressing results, with multiple sources confirming that SGM experiencing intersecting marginalisations have -and do- consistently experience poorer health outcomes [58, 59]. Evidently, the medical field has a distorted view of SRH care for women due to systematic oversight from research through to clinical settings, which is likely further compounded for SGM. In recent decades, attention and subsequent outrage has been building in Australia to address systemic barriers as we court gender parity [60–63]. One Australian scoping review [55] graded articles published between 2009 and 2019 and discovered that misogynistic attitudes continue to permeate medical narratives. Another study [64] assessed the Australian clinical guidelines of the last decade for inclusion of sex and gender, and found that of 65 clinical practice guidelines concerning sex, 12 included gender relevant practises, while only four offered definitions. This reflects a concerning but widespread trend of using the terms “sex” and “gender” interchangeably, which typically assumes cis-heteronormative features and further marginalises SGM, but also skews our understanding of problems encountered by the broader population. For instance, health information systems that only offer a single field for the record of sex or gender, functionally eliminate the trans SRH experience from the record. Since clinicians have no way to systematically record this, public health researchers have no way to aggregate the data for the detection of biological trends specific to the trans experience. In this context, apathy toward social differences and the desire for binary categorisation ignores scientific constructs, and further mystifies the biological phenomena of STIs.

In this review, ‘sex’, ‘gender’, and ‘sexuality’ bear different implications regarding socio-sexual behaviours and access to SRH care. Whilst both sexes can harbour diverse microbiota, the assigned male at birth (AMAB) genital microbiome is described as relatively simple and stable compared to its more structurally dynamic assigned female at birth (AFAB) counterpart, and research into SGM microbiomes is scarce [65]. Herein, we choose to focus on the vaginal microbiomes due to its complex micro-ecology, lack of research in STI evolution, and the collision of these biological factors with social factors which influence the SRH care of AFAB and SGM individuals. Not all vulval and vaginal environments belong to AFAB people, nor do all AFAB people identify as women. Hence, the physiology of the various vaginal microenvironments is discussed in detail as the microbial ecology of each is unique. Several authors have highlighted that the lack of nuanced understanding across these niches creates barriers to accessing adequate care for patients outside of cishet norms [57, 66–69]. Given that social and physiological contexts are equally relevant in directing patient care at the clinical level, this raises serious concerns and underscores a clear area of need for future research.

### Sexual networks play a role in shaping microbiome composition

Despite the central importance of social context in STI transmission, we have a very limited understanding of sexual networks, and poor data collection represents a major constraint amongst sexual health professionals [57, 70–72]. Historically, HIV and other STIs were frequently framed as predominantly affecting GBMSM, particularly evident in the early stages of the AIDS crisis when the disease was initially termed Gay-Related Immune Deficiency (GRID) [1–3]. This initial framing, reinforced by social stigma and early public health messaging contributed to the common misconception that transmission was confined to GBMSM sexual networks as opposed to broader, more interconnected populations [10, 73]. Whilst this has been proven erroneous, heterosexual transmission dynamics remain comparatively under-researched [11]. Recent research has indicated that there is considerable ‘bridging’ of STI pathogens from GBMSM to ‘heterosexual’ networks, despite potentially controversial codification [39, 74]. This has important implications for surveillance, suggesting that observed epidemiological patterns may be shaped by detection practises and historical policies as well as underlying biology. Consequently, the complexity of STI transmission and its true incidence may be obscured when analyses prioritise incidence without sufficient metadata or investigation of biological mechanisms driving pathogen transmission. This limitation extends beyond epidemiology and affects our understanding of STI micro-ecology. By failing to consider the biological and environmental contexts in which pathogens operate, correlations may be misinterpreted or misattributed. Incorporating ecological perspectives – including metabolic and environmental requirements of pathogens - would aid our understanding of pathogenesis and transmission risk. For example, one environmental study considered that surfer exposure to wastewater in the United Kingdom may be linked to greater anal colonisation by AMR pathogens than their non-surfing counterparts [75]. This illustrates how context-specific exposures can also shape risk in ways not captured by traditional surveillance, and underscores the importance of accounting for context when examining AMR-STIs. Applying similar ecological and network-informed considerations to STI research - which account for the diversity of sexual practises, microenvironments and transmission contexts – could substantially improve our understanding of AMR-STI evolution and emergence.

Most importantly, treatment does not occur in a vacuum but may indeed bear some responsibility for differences in AMR-STI emergence across SGM groups. Because research has traditionally treated GBMSM as a closed community, so too have clinicians and policymakers in directing resultant STI treatments. In this way, therapeutics may act as selective pressures, guiding STI evolution within specific subgroups given their selective application in clinical settings. If GBMSM are considered an ‘at risk’ group due to their higher reported incidence of STIs and are therefore selected for additional screening compared to their women-who-have-sex-with-women (WSW) counterparts, then a cycle of increased detection and treatment is bound to emerge, skewing the true incidence and reaffirming a heightened incidence rate among GBMSM. Further, sexually active, gay or bisexual trans men are GBMSM in sexual identity, network and practise, although they possess AFAB anatomy, and a vaginal microbiome (VMB) composition which is not well understood at present. These men are perhaps at increased risk of contracting AMR-STIs due to their GBMSM risk group as highlighted in Australian STI management guidelines [76]. However, due to their AFAB status, they are not eligible for doxycycline prophylactic treatment (Doxy-PEP) received by their cisgendered partners [77]. Doxy-PEP is widely thought to select for AMR in gonococcal infections [78, 79], hence placing these trans GBMSM at greater risk of contracting an AMR-STIs. Trans women who were assigned male at birth (AMAB) face the same issue of Doxy-PEP exclusion in Australia despite their consistent mention in pioneering guidelines overseas [80, 81]. These issues are compounded by the inability of public health systems to detect biological phenomena specific to SGM individuals. Subsequently, SGM individuals must be seen, screened and treated on a case-by-case basis, which is adequate for individual case resolution, yet is challenging for the broader community to learn and benefit from a wider range of cases. This obfuscates the phenomena experience by SGM and acts as a barrier to the implementation of a universal standard of care for STIs within these ‘higher risk’ groups.

There are several key things to be learned from pairing good sexual network data with STI incidence and surveillance: (i) which transmission pathways select for AMR emergence?; (ii) does this present a heightened risk for a particular community or demographic?; (iii) are some microenvironments more prone to colonisation or infection?; (iv) do specific sexual practises promote risk of infection, and if so, what education and preventative approaches can be implemented?; (v) does sex, gender, or sexual network act as the primary driver of STI evolution? Whilst these relevant questions each deserve impartial and thorough investigation, a perspective informed by microbial ecology would proffer that (i) sexual networks dynamics are likely the drivers of transmission, and (ii) the niche microenvironment would determine organism fitness and therefore evolution through gene expression.

## The vulval and vaginal microbiomes

In clinical settings, the terms ‘vulva’ and ‘vagina’ are often used interchangeably despite being different organs, which can lead to confusion [44], hence despite the emergence of the term ‘vulvovaginal’ in recent scientific literature, it is non-preferred. We use the terms ‘vagina’ to refer to the internal genital tract from vaginal opening to the cervix, and ‘vulva’ to refer collectively to the external genitalia surrounding the vaginal opening including the labia majora, labia minora, clitoris, and several less commonly known anatomical features [44, 82].

Research on the vulval microbiome is emerging, and has benefited from recent investigative efforts, with microbial composition suspected to play a key role [83]. Healthy vulval microflora represents a distinct micro-ecological niche which is more diverse than in the vagina, with microbes of vaginal, urethral, cutaneous, or intestinal origin evident [83, 84]. Data remains limited, though it is theorised that the vulva constitutes an ‘inward-facing gradual transition zone’, wherein microflora transition from cutaneous commensals to microflora, reflecting vaginal and intestinal influences [84]. This makes sense from a micro-ecological perspective and affirms previous suggestions that vulval flora may affect pathogen proliferation [83]. Furthermore, while cosmetic surgeons advocate that differences in vulval anatomical structure influence infection rates [85], this claim remains contentious with little supporting evidence [86]. Although VMB composition is likely linked with vulval microflora and may contribute to STI acquisition (especially in the context of penetrative sex), the field remains comparatively under-studied. One common thread is that diversity exists both within and between individuals [87], making research efforts challenging. Indeed, defining ‘women’ proves relatively difficult due to the overwhelming and inevitable level of change that AFAB people experience throughout a lifetime. Even among cis women, the shifting nature of the VMB results in highly individualised needs, which are dependent on personalised circumstance (age, hormonal status, sexual engagement, etc.). Here, we describe relevant biological factors implicit within hormonally and physiologically differentiated microenvironments to elucidate the relevant micro-ecological factors. Perineal health is also relevant to VMB composition given its proximity to faecal contaminants [44], although is not the focus of this review.

### Vaginal microbiome

**Fig. 2:**
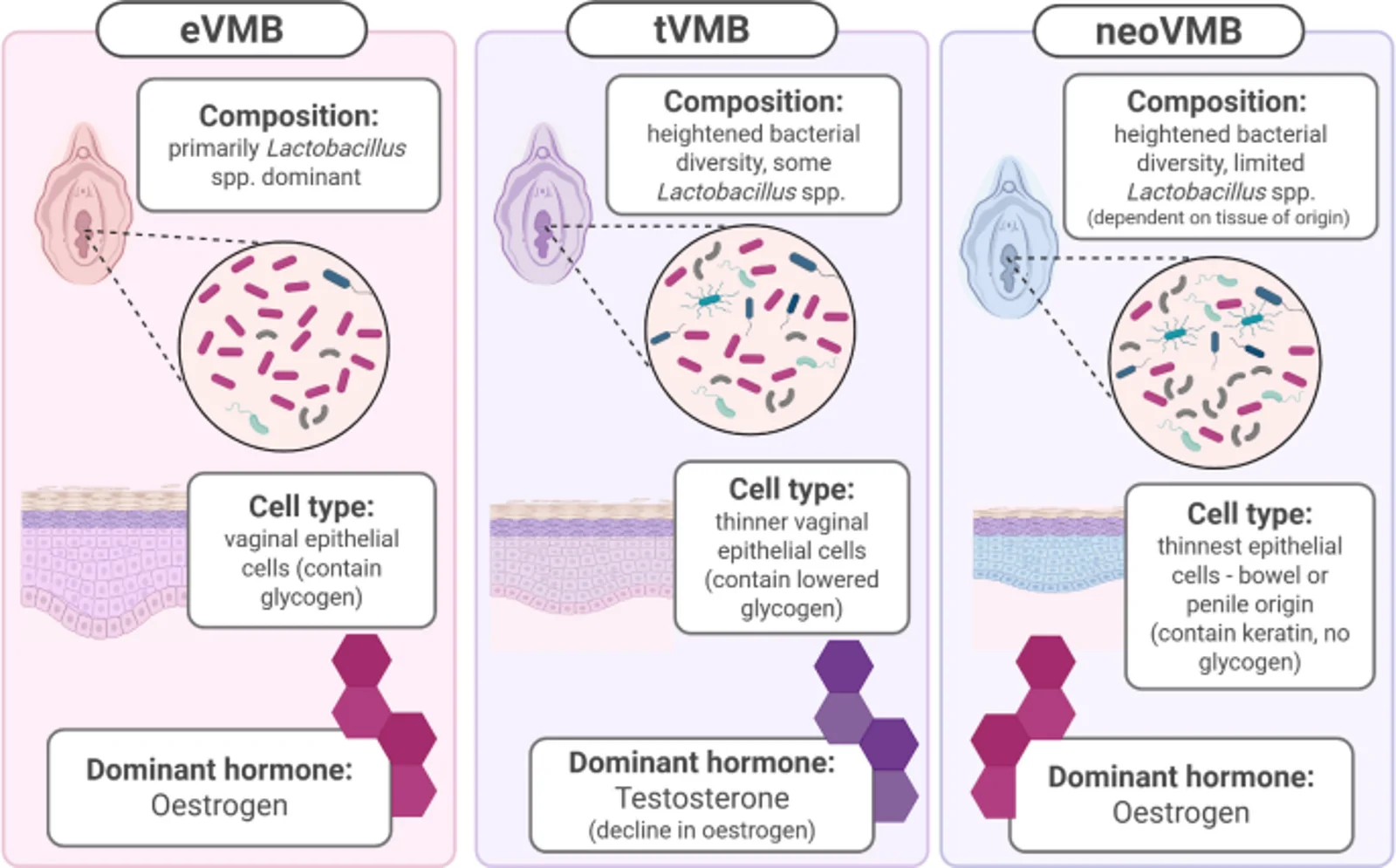
Summary of key vaginal microbiome and microenvironment features. Features of three vaginal microbiomes are summarised. (i) the eVMB occurs in an oestrogen-dominant microenvironment with vaginal epithelium containing glycogen (serving as a metabolic energy source for resident microbes). eVMB composition is typically Lactobacillus spp. (pink) dominant, though this is influenced by factors such as race, age, and menstrual milestones. Gardnerella vaginalis (grey) and other microbes are also common in the eVMB. (ii) the tVMB occurs in a testosterone-dominant microenvironment with thinner vaginal epithelium and lowered glycogen. tVMB composition is prone to heightened bacterial diversity compared to the eVMB. This is similar to both the post-menopausal VMB and transitional/BV-associated compositions, with a higher relative abundance of G. vaginalis (grey). (iii) the neoVMB occurs in an oestrogen-dominant microenvironment with the thinnest epithelial lining. As this lining is typically surgically constructed from bowel or penile origin, its epithelial cells contain no glycogen, explaining the low Lactobacillus spp. abundance and heightened bacterial diversity of the neoVMB. The neoVMB is directly influenced by microbes endogenous to the lining tissue, hence gut and skin-associated bacteria are commonly observed. VMB- vaginal microbiome; eVMB – oestrogen-dominant VMB; tVMB – testosterone-dominant VMB; BV – bacterial vaginosis; neoVMB – neovaginal VMB. Created in BioRender [90]

Vaginal microenvironments occur across a spectrum with relatively unique microbial compositions (see Fig. 2). Speculation as to how different factors might affect the inhabitant microbial ecosystems is important, although definitive results remain elusive. Microbial composition norms are difficult to establish in vaginal microenvironments due to the complex interplay of many confounding variables. Hormones, menstrual cycles, diet, hygiene practices, sexual behaviours, immune function, pregnancy status, and STIs all impact VMB ecology. Simply put, adequately powering studies to consider all of these factors is incredibly resource intensive. No singular study has successfully addressed all these factors to date, although several have made significant inroads in understanding each component. Research into the VMB has been of keen interest to microbial ecologists in recent years, especially due to direct contrasts with gut microbiome dynamics. In the gut microbiome, heightened microbial diversity indicates health, whereas heightened diversity of vaginal flora can signal dysbiosis or pathologies [26, 88], with the exception of pregnancy, which exhibits *Lactobacillus crispatus* dominance. Although several efforts to categorise VMB composition have been made to date, no universal definitive causal link has been reproducibly established between VMB composition and clinical symptom presentation.

Here is what we do know: microflora in the VMB is known to fluctuate due to both internal and external factors, which respectively refer to intrinsic biological factors outside of host control and behavioural factors that are within host control [61]. Internal factors include age, hormonal shifts (menarche, menses, pregnancy), and infections, whereas external factors include antibiotic use, sexual intercourse, hormonal contraceptives or replacement therapies, and hygiene practises [44, 83, 89]. It is worth noting VMB composition studies have traditionally preferred a ‘healthy’ state without real consideration of STIs, dysbiosis, or subsequent AMR pathways, although research into bacterial vaginosis has been expanding into this space. Studying the VMB in an unbalanced or infected state may assist in our understanding of how resistance is conferred within these environments. Investigating how highly mutable organisms engage in rapid AMR acquisition (such as *Neisseria gonorrhoeae;* henceforth Ng) may be particularly valuable given its development of resistance to all available treatments. In the global context of increasingly resistant pathogens, all potential AMR development pathways should be thoroughly examined in hopes of discovering underlying mechanisms and preventative approaches. This section will explore the different vaginal microenvironments, which could be considered internal factors in contributing to VMB composition, as well as some external factors known to impact VMB health.

#### Oestrogen-dominant Vaginal Microbiome (eVMB)

Like other mucosal sites, the vaginal mucosa forms a first line of defence, whilst the oestrogen-dominant VMB (eVMB) maintains dynamic homeostatic equilibrium [27], protecting against urogenital pathogens [91]. Despite not being produced by the vagina, hormone-regulated cervical mucus is rich in glycoproteins termed ‘mucins’, which protect vaginal epithelial cells from pathogenic threats [92], nourish microbiota [93], and modulate host-microbe interactions [94]. Vaginal epithelial cells continuously renew and differentiate before achieving full maturation [95] similar to skin epithelial cells. The vaginal epithelial layers are comprised of an active basal layer (*stratum basale*), suprabasal layer, and a superficial layer of flattened cornified cells called the ‘*stratum corneum’* (SC) [96], which forms the primary interface between host and pathogens. It is important to note that these SC cells lack the biological machinery required to produce pathogen defence responses, making them generally more permeable [96]. This means that these epithelial cells that form the vaginal lining and a physical barrier are more permeable to both pathogens and endogenous microbes. Furthermore, vaginal epithelial cell differentiation and maturation are regulated by the sex hormones oestrogen and progesterone, which respectively increase or decrease epithelial thickness, thereby controlling its structural integrity and ability to function as a barrier [97, 98]. Consequently, vaginal epithelial structure responds to hormonal and environmental influences across the menstrual cycle [26], and throughout life (i.e. puberty, menopause, or uterine prolapse) [96]. From a micro-ecology perspective, the oestrogen-dominant vaginal microenvironment challenges its inhabitants to be either inert or counter-responsive to hormonal influences and a dynamically changing structure to survive.

In the traditionally healthy eVMB, *Lactobacillus* spp. (Lb) dominate and support health through several mechanisms [99]. Firstly, Lb metabolises glycogen from shed vaginal epithelial cells to produce lactic acid, which subsequently lowers vaginal pH, favours Lb proliferation [100–103], and inhibits pathogen dominance [104–110]. Secondly, Lb outcompetes other microorganisms to bind to the surface of vaginal epithelial cells and secrete antimicrobial compounds [44, 83, 111, 112]. Additionally, Lb has immunomodulating, antioxidant, and anti-biofilm properties [99] which also contribute to their success inhibiting pathogen growth. Sex hormones have been found to regulate the abundance of Lb in the vaginal mucosa, with the menstrual cycle clearly seen to directly affecting microbial diversity [113–115]. This finding is concordant with the evidence that pregnancy is associated with an increase in sex hormones as well as a stable Lb-dominant eVMB with low diversity [116, 117], whereas the postnatal eVMB shifts toward non-Lb-dominance [117]. Hormonal contraceptives have been observed to impact Lb-dominance in some studies [115, 118], although they were found to be insignificant in others [114], and may differ due to hormone composition and placement [113]. As with glycogen-hormone relationships, the complex dynamic between endocrine effectors and eVMB composition warrants further investigation [119]. Taken together, it is apparent that Lb are important for the maintenance of eVMB composition, although the mechanisms supporting health as well as host-Lb interactions require further investigation to determine exactly how, -and if- similar effects can be synthesised through alternative VMB compositions.

It can be argued that Lb-dominance is a type of mutualism, especially since host defence systems are primed to support this relationship in both structural properties and metabolic provisions. Whilst both vaginal and epidermal epithelial cells are loosely connected by weakened intercellular junctions, one key point of difference is that cornified SC vaginal cells are abundant in glycogen stores [26, 96], theoretically selecting for bacteria with glycogen-metabolic capabilities, such as Lb. Frequent exfoliation of the SC epithelial layer is suspected to play a role in host-defence by sloughing ‘decoy’ cells at a heightened rate; this ‘tricks’ pathogens into binding to cells which are subsequently excreted, and disallows STIs from penetrating the body [96]. Additionally, decoy-sloughing doubles the provision of free glycogen through cell degradation, thereby further promoting Lb-dominance by increasing free glycogen. Glycogen storage also appears to increase vaginal epithelial cell permeability to both microbes and immune defence mediators, and is suspected to foster endogenous vaginal flora to maintain a low pH [96, 101], supporting that Lb might be selected for as an extension of VMB defence. Whilst these structural features all appear to select for Lb dominance, sex hormones also contribute.

Oestrogen has been implicated in immune response modulation [119–121], and also promotes glycogen.

production within the vaginal mucosa [96, 122, 123], indicating it likely plays a role in selecting for Lb dominant VMB composition. Sex hormones seemingly bear an influential, albeit complex, role in glycogen availability throughout the menstrual cycle [26, 124], where heightened levels of free glycogen are clearly correlated with Lb-dominance [100–102]. From the inhabitant microbe perspective, host glycogen storage and regulation are key metabolic drivers of evolutionary fitness, hence microbes which can metabolise glycogen are likely to thrive. Finally, whilst several mechanisms which promote Lb-dominance and subsequent health have been discovered, not all ‘healthy’ VMB compositions are Lb-dominant, nor is there a distinct formula predictive of SRH outcomes, with study design paradigms often subject to valid criticism.

##### VMB composition and the influences of race and ethnicity.

A key contention in VMB research is the influence of race and ethnicity, which appear to be closely correlated with VMB composition. The earliest study of VMB composition investigated North American women of reproductive age. The authors leveraged 16S sequencing to create a typology of five ‘community state types’ (CSTs) based on distinct clusters of microbial taxa and dominance [125]. Four CSTs exhibited Lb-dominance (CST I, *L. crispatus*; CST II, *L. gasseri*; CST III, *L. iners*; CST V, *L. jensenii*), whilst CST IV was more diverse with a high presence of strict anaerobes [126]. Although this CST was overrepresented in Hispanic and African-American women as compared with their Caucasian and Asian counterparts, it was not necessarily concordant with symptoms or health status [125]. However, subsequent research has indicated that CST classifications are wanting of finer resolution, and subtypes have been offered by larger scale studies to better distinguish CST subvariants, as well as tools for standardisation between studies [127–129].

Another tact which considers the importance of specific species has also yielded progress, but no clear conclusions. Globally, studies have demonstrated the prevalence of *Lactobacillus Iners* (Lb-i), *Lactobacillus crispatus* (Lb-c), and *Gardnerella vaginalis* (Gv) in VMBs of reproductive-aged women from every population so far, including those from North [129] and South American [130], European [131], African [132, 133], and Asian [134, 135] populations. It is important to highlight that not all healthy eVMB compositions are associated with Lb-dominance, although composition appears highly dependent on ethnicity or race [26, 136]. This is further compounded by the finding that Lb-non-dominance does not necessarily indicate poor health, especially in non-white populations [137, 138]. Furthermore, not all Lb are associated with favourable SRH outcomes [125, 127]. Despite the lack of a definitive health-predictive characterisation of VMB composition, some things are becoming clearer. Recent research indicates that the lactic acid mechanism is retained even in non-Lb-dominant ecosystems, though microbial composition may also be more variable than previously thought [83]. Ultimately, characterisation of the driving factors of racial and geographic differences in VMB composition remain elusive to date. However, socio-contextual factors have been repeatedly implicated and warrant further interrogation (see reviews [26, 139, 140]).

Several findings indicate that angling biological research to better quantify relevant social factors may yield clearer inferences about VMB composition. One cross-sectional study found that VMB profiles across multiple racial demographics shifted towards Lb-dominance during pregnancy, with the VMBs of African and Hispanic women changing significantly to achieve Lb-dominance [141]. The authors highlighted the importance of confounding factors including age and socioeconomic status, which could not be decoupled from race [141]. Another study identified education and socio-economic status as important co-factors in VMB composition, noting a positive correlation with Lb-c dominance [142]. This tells us that race and socioeconomic status are both important factors which likely both contribute to shaping VMB composition. In 2022, this was explored by an investigation of preterm birth, VMB composition and race [136] which reported several key findings: (i) the VMBs of African-American women exhibited high species diversity, with high Lb-i but low Lb-c abundance, (ii) no significant differences in VMB composition were found among women who douche, and (iii) spontaneous preterm birth was associated with low Lb-c abundance, but species diversity and Lb-i did not significantly contribute. The authors noted strong correlations between maternal factors (poverty, education, marital status, age, douching, race) and VMB composition, suggesting that race may be a proxy for socially driven factors impacting VMB structure which require further investigation alongside the impacts of douching [136]. Taken together, these findings underscore the importance of defining the many different social factors which coincide with race and ethnicity and may shape micro-ecological findings. One recent study attempted such a lens. In 2023, a group investigating the VMB impact of socio-contextual determinants found that women experienced aspects of their residential neighbourhood context differently based on their individual socioeconomic context, with joint-effects of these factors impacting VMB composition in a potentially synergistic manner [143]. Stress appeared to have a dysregulating influence over the VMB, yet the mechanism of action is not yet understood, with investigations into the role of cortisol remaining inconclusive [144–146]. Considering the effect of stress, some authors postulate that the disparity of Lb-dominance observed across racially diverse groups and heightened bacterial diversity observed in non-white cis women may be due to unmeasured discrimination factors such as systemic racism, psychosocial stress and low socio-economic status [142, 146, 147]. In summary, social factors are deeply important in shaping VBM micro-ecology, though future investigations should aim to understand how these mechanisms function.

Taken altogether, several important points have emerged which deserve clarification. Firstly, there are observed differences in VMB composition across racially diverse groups. Whilst the driving mechanisms are yet to be defined it seems likely that racial characterisation may act as a proxy for confounding underlying socio-contextual determinants. It would be unsurprising if a similar proxy was in effect for other marginalisations, including sex worker status or sexual orientation. For instance, douching has been associated with non-Lb-dominance in both African-American and Caucasian women [136], and may represent a self-sought resolution to symptoms if an individual is experiencing multiple barriers to healthcare access, such as is common for African-American women. Secondly, these different VMB compositions are not a universal indicator of poor health. However, Lb-dominance does demonstrate protective qualities, and epidemiological studies have linked non-Lb-dominance with increased risk of STI acquisition [132, 148–150] and spontaneous preterm birth [127, 151–157], indicating it may be a risk factor. From a micro-ecological perspective, spheres of influence on the individual likely determine some portion of the vaginal microenvironment and cannot be extricated as potential drivers of VMB composition. If race or ethnicity act as proxies for culturally influenced diet or hygiene factors, it is likely that VMB compositions would be similar. If they also act as proxies for lack of access to SRH care or persistent oversight by medical professionals, which creates a need for self-medication using old or inappropriate prescriptions, then the emergence of AMR-STIs within racial or ethnic subgroups is not a factor of race or ethnicity, but evidence for biological phenomena driven by social influence. This theory is similarly applicable to contexts of age and SGM, underscoring the need for well-designed, culturally relevant STI research which adequately considers social factors.

##### Prepubescence, menarche, pregnancy, & menopause

As outlined earlier, sex hormones can influence the vaginal microenvironment and therefore VMB composition. Menstrual milestones also play a key role, although pre-pubescent and post-menopausal women receive little attention relative to the eVMBs of ‘reproductive-aged’ women (15–45 years). Whilst several studies report findings correlating VMB composition with age, this is likely to operate as a proxy for menstrual milestones, hence we have addressed them directly.

**Prepubescence.** Research indicates that healthy VMB composition of prepubescent individuals is highly diverse with typical dominance of various aerobic and anaerobic bacterial species, but low abundance of Lb, Gv, and *Prevotella bivia* [158]. The vaginal microenvironment is notably different during prepubescence, with a neutral or slightly alkaline pH, absence of cervical mucus, and thinner vulvar and vaginal epithelium owing to lower oestrogen [159–161]. One study found that converse to eVMB composition, heightened diversity is indicative of good health in the prepubescent VMB, whilst lowered diversity and dominance of specific bacterial species is associated with vulvovaginitis [162]. The prepubescent vulval and vaginal microenvironments combined with the poorer hygiene habits of children presents a favourable situation for enteric and respiratory pathogens, hence they are more common in prepubertal vulvovaginitis [160] than their reproductive aged counterparts. As oestrogen increases with the onset of puberty, the vulval and vaginal microenvironments transition toward eVMB and exhibit increased glycogen production, leading to Lb-dominance and an acidic pH well in advance of menarche [144, 163–165]. However the vaginal pH remains higher following menarche than is considered typically healthy in reproductive aged cis women, indicating that Lb reach adult counts later in development [163]. Whilst it is likely that the prepubescent VMB remains underrepresented in research due to ethical quandaries and limited clinical presentation, our depth of understanding regarding VMB micro-ecology could benefit pubertal girls presenting with uncomfortable symptoms and could assist in clarifying racial disparities.

**Menarche**. Again, whilst more investigation is required, some things regarding the effect of menstrual phases on VMB composition are clear. It is well understood that in women of reproductive age, oestradiol elevation promotes glycogen accumulation and subsequent Lb colonisation during the several menstrual phases, whereas oestradiol reduction during menstruation is correlated with low Lb counts and increased bacterial diversity [114]. Hormonal contraceptives do not ‘interfere’ with natural fluctuations in eVMB composition [114, 166], although have long been indicated to have a stabilising effect [118, 167–171]. This means that in line with other findings, sex hormones are implicated in shaping VMB micro-ecology, however several confounding variables warrant further investigation. The Isala citizen-science study conducted in Belgium analysed 3,345 self-collected eVMB samples alongside detailed questionnaires; they found eVMB composition strongly correlated with menstrual cycle phase, age, and childbirth status [171]. They also noted differences in eVMB composition between the participants who identified as WSW and their heterosexual counterparts, with associations between relative abundance of certain taxa and recent sexual intercourse [171]. This lends credibility to the idea that eVMB composition is influenced by not only internal microenvironmental factors, but also by engagement with sexual networks. Together, these findings highlight the dynamic nature of VMB micro-ecology both within and between individuals. Interestingly, this is converse to findings regarding pregnancy.

**Pregnancy**. Pregnancy is associated with an increase in sex hormones and a stable Lb-dominant eVMB that is low in bacterial diversity [116, 117]. This is protective against both infection and preterm birth, and differs from the non-pregnant eVMB, which is relatively diverse in comparison [172–174]. Several studies have confirmed that the postnatal eVMB sees a shift toward heightened bacterial diversity and non-Lb-dominance [117, 171], with eVMB disturbance persisting for up to one year post-partum [175]. The Isala study further demonstrated a significant association between women who had been pregnant and a reduction in several Lb as well as an increase in taxa involved in initial gut colonisation of babies [171]. Other related effects, such as decreased pelvic floor muscle function, have been associated with disrupted eVMB composition, indicating that physiological changes resulting from birth may also play a role in composition [162, 176]. Further elucidation of SRH protective mechanisms may come from investigations into changes in the vaginal microenvironment considering the eVMB in association with conception, pregnancy, and post-natal changes.

**Menopause.** The vulval and vaginal microenvironments again profoundly change during menopause. Menopause is marked by a decline in oestrogen, thinning vaginal epithelium [177], lowered glycogen and Lb concentration [101], increased vaginal pH [178], and heightened bacterial diversity in the eVMB (see reviews [26, 119, 158, 179, 180]). Cervical mucus production, composition, and spread also change alongside declining sex hormones [181, 182]. Menopausal physiological and anatomical changes to the vaginal microenvironment and VMB can lead to vaginal dryness and discomfort symptomatic of vulval and vaginal atrophy (VVA) [183] and genitourinary syndrome of menopause (GSM) [184]. One study demonstrated that VVA is associated with CST IV-A and the low relative abundance of Lb [183]. It is worth highlighting that the post-menopausal VMB composition is characterised by high concentrations of anaerobic bacteria and therefore are similar in composition to the prepubescent VMB [163, 185], indicating that an increase in SRH complaint of this milestone group could be associated with sexual networks or function (or an increased awareness and capacity to self-advocate). Research describing the post-menopausal VMB of non-white women is lacking, although differences in Lb dominance between African-American and Caucasian cis women have been reported [186]. Hormonal replacement therapy (HRT) is a primary treatment option for VVA and GSM, and has been proven to increase Lb abundance and subsequently restore vaginal health [187–191]. Whilst research is increasing in this area, it is important to monitor the literature to identify ways we can synergistically benefit multiple groups. For instance, the post-menopausal VMB is quite similar in composition to the testosterone dominant VMB (tVMB) which can lead to similar complaints from AFAB trans men. However, we must consider that if research is lacking and post-menopausal cis women are not well-catered to, then the experience of AFAB trans men is likely even more disillusioning, and complaints are likely underreported.

#### Testosterone dominant VMB (tVMB)

Trans men and non-binary AFAB individuals may also have vaginal microenvironments and require gynaecological care, though the micro-ecology of the tVMB is not yet well understood. Testosterone therapy is a proven effective treatment option for individuals aiming to promote the development of ‘masculine’ secondary sex characteristics. However, oestrogen suppression may lead to symptoms observed post-menopause including vaginal epithelial thinning, high pH, and low Lb colonisation reminiscent of GSM [192–194].Transmasculine AFAB individuals undergoing testosterone therapy commonly experience dryness, irritation, and bleeding with vaginal penetration [192, 195]. Common in post-menopausal cis women, these symptoms are typically treated with localised oestrogen, although efficacy and VMB composition effects remain poorly documented in the tVMB [195–197]. Recent research has found the tVMB shifts toward heightened microbial diversity similar to the post-menopausal VMB, although Lb loss is more pronounced, and sexual activity does not appear to have the same influence over the tVMB [193]. Although limited in sample size, such findings regarding low Lb abundance support earlier reports [197]. These two studies form the only tVMB assessments to date, and report that despite displaying similar trends to post-menopausal VMBs, the tVMB is distinctly characterised by intestinal taxa [193, 197]. The authors are unaware of any large-scale study to date investigating sexual network engagement with tVMB composition, although this would be a most welcome addition to the field.

It is important to note that many transmasculine AFAB individuals interrupt testosterone therapy to fall pregnant [198]. Impacts to tVMB composition and subsequent SRH outcomes require further investigation. Given the difficulty which SGM experience in accessing gender affirming and SRH care, it is likely that attitudes to oestrogen treatments are hesitant in testosterone users, and bear influence on treatment uptake. Additionally, if there is no normalised system of recognition for SGM in healthcare settings, then this may act as a further barrier to accessing SRH care. Further, this knowledge gap forces SGM including trans men to rely on unofficial sources for experiential information, which puts them at increased risk of harmful misinformation [199–202].

#### Neovaginal microbiome (neoVMB)

Trans women and AMAB individuals who have undergone gender-affirming vaginoplasty also require clinical care with aptly trained gynaecologists and gender specialist surgeons [203, 204]. Clinical goals of feminising genital surgery often include elements such as aspiring to achieve concordant neo-vulvar anatomy, moist and hairless vaginal canal with adequate depth and width for penetration, erogenous sensation, and minimal maintenance [205]. The surgical gold standard of feminising genital surgery is penile inversion vaginoplasty to create a ‘neovagina’, which requires multiple surgical steps [195, 206–211]. Typically, preserved penile tube skin is inverted and used as the neovaginal canal, with additional scrotal skin grafts used to augment length [212–215]. When penile and scrotal skin are insufficient to achieve desired function, revision surgeries may augment length by using bowel pedicle flaps from portions of the colon, skin flaps from the thigh or lower abdomen, peritoneal flaps, or urethral mucosa [see review [195]]. Neovaginal linings are most commonly from either penile scrotal flaps [207, 208] or rectosigmoidal colon bowel flaps [207]. This is important because as vaginal epithelial cells shape the VMBs of AFAB individuals, cells which line the neovaginal canal likely dictate the composition of the neovaginal microbiome (neoVMB) and subsequent function [195].

Like the vaginal epithelial layer, penile skin constantly renews through basal cell proliferation, although it is thinner than pre-menopausal vaginal epithelium [194, 216], and its cornified cells are filled with keratin bundles as opposed to glycogen [217, 218]. Hence, shedding of neovaginal epithelial cells likely fosters a different microbial community to the eVMB and tVMB, which may explain a lower Lb and higher cutaneous species presence in neoVMB composition [219–222], especially when penile skin has been used for neovaginal surgery. One investigation into penile skin derived neovaginal epithelium confirmed that even long-term oestrogen recipients did not demonstrate glycogen production, lending credibility to the expectation that Lb are unable to be supported in the neoVMB [195, 223]. In contrast, the sigmoidal colon epithelium is highly distinct from both vaginal and penile epithelium and contains both goblet and paneth cells, which produce mucus and antimicrobial peptides respectively [195]. Although studies are limited, it appears that the neoVMB is defined by microbiota which colonise the relevant tissue of origin [195]. Taken together, clinicians should account for variation when investigating neovaginal complaints as patients are likely to benefit from personally customised treatment plans and courses. Despite increased investigation into the neoVMB in recent years, a clear understanding of optimal microbiota versus those associated with clinical symptoms or increased STI acquisition is yet to be defined.

Traditionally, neoVMB research has been limited to cis-centric investigations to identify Lb or STI pathogens of interest [195, 224–229]. Given the neo-VMB is heavily influenced by tissue origin, research designs which compare neoVMB composition with eVMB composition are potentially ill-fated. Recent research has taken a different approach yet warrants further extension. One study combined proteomics and 16 S rRNA sequencing to investigate the neoVMB of a small sample of vaginoplasty recipients (*n* = 5; 4 penile inversion, 1 sigmoid) [230]. Whilst power-limited, this study highlighted several key findings to direct future neoVMB research: (i) low Lb abundance, (ii) penile skin lined neoVMBs had a high prevalence of *Prevotella*, *Peptostreptococcus*,* Peptoniphilus*,* Corynebacterium* species, like the post-menopausal VMB and tVMB (and the foreskin of the uncircumcised penis), (iii) sigmoidal neoVMB was distinct and contained taxa common to the gut microbiome [230]. Another study (which has yet to undergo rigorous peer review) characterised the neoVMB and cytokines of 47 trans women alongside self-reported sexual behaviour and symptoms [231]. The authors reported low Lb-dominance, as well as four distinct bacterial taxa clusters with unique immune profiles, suggesting interplay between bacteria and neovaginal tissue, and supporting the current dominant theory that tissue used in neovaginal construction strongly influences neoVMB composition [231]. Although these findings are yet to be accepted, the authors offer the first clear insight into ‘optimal’ neoVMB composition and note a lack of correlation between reported symptoms and composition, similar to issues encountered in eVMB dysbiosis research. Similarities between the tVMB, neoVMB, and post-menopausal VMB indicate a need for further research into these non-oestrogen dominant VMBs which may yield synergistic benefits to several communities. Common to all research to date is the need to create clinical guidelines to address complaints pertinent to VMB composition.

### Behavioural factors of influence in the VMBs

Having discussed the relevance of sexual networks, distinct microenvironments, and various VMB compositions, it is pertinent to now direct our attention toward confounding behavioural factors within host control and universally changeable at the individual level. Smoking [232–234], diet, and gut-issues [235] are also known to impact VMB composition through various mechanisms. Whilst these technically constitute external factors, they are also part of a broader socio-cultural context as opposed to distinctly individual, hence are not discussed here. Specifically, we provide direct attention to hygiene, aesthetic and antibiotic consumption practices.

#### Hygiene practices

Hygiene practices provide an important context for VMB composition from wiping strategy through to douching, which may be influenced by sociocultural contexts. For instance, douching has a known association with bacterial vaginosis (BV), although it is unclear whether BV symptoms lead to douching, or douching is causative of BV [136, 236–238]. Given the association of BV with adverse SRH outcomes such as spontaneous preterm birth, a recent study highlighted the importance of considering relevant social context (in this case, self-reported race) [136].

Douching appeared to normalise the typically distinct VMB compositions of African-American and Caucasian women. However, this only appeared to have an adverse effect on SRH outcomes of Caucasian women, whose VMB composition exhibited a more dynamic change following douching [136]. This could perhaps be supported by previous findings that African-American women are more likely to engage in vaginal douching [239, 240], suggesting a compounding interplay between VMB dynamics, sociocultural context, and health outcomes. Given our increasing understanding that microbial colonisation is often determined during vaginal birth, this raises an interesting biosocial question. Has the ecological composition of eVMBs in African-American women adapted to accommodate this hygiene practise over time, resulting in the prevalence of the distinct CST IV within this specific population group, does CST IV drive symptoms and therefore douching as self-medication, or is a complex alternative explanation more apt? Further investigation in VMB dynamics alongside symptoms and douching behaviour would provide important information in this regard.

Other hygiene practises are also likely important in shaping VMB composition. Perianal colonisation and wiping habits may also contribute to VMB disruption, with UTIs, BV, and yeast infections commonly arising from passive microbial transfer from the anal opening along the perineum i.e. wiping from back to front [241–244]. Habitual bidet use is also thought to facilitate the transfer of faecal and subcutaneous bacteria toward the vulvovaginal anatomy, resulting in dysbiosis [245, 246]. This has far-reaching implications for cultural regions such as Japan and Europe, which routinely use bidets.

#### Aesthetic practises

Aesthetic practises which alter the vaginal micro-environment warrant examination when considering how VMB micro-ecology is shaped. ‘Beautification’ practises targeting vulval and vaginal anatomy have become increasingly fashionable despite their relatively understudied nature and potential impacts on intimate health. These range from practices such as pubic hair removal through to surgical intervention such as labiaplasty, and have been thoroughly reviewed elsewhere [see [44, 83]], as has the detrimental psychological impacts of extensive marketing toward improving feminine appearances [see [247–249]].

Pubic hair removal has become increasingly prevalent, especially among young, sexually active women [250, 251]. However, practices differ between cultural demographics such as race [251] and sexual orientation [252]. As a protective feature of the vulval and vaginal anatomy, pubic hair removal may increase susceptibility to pathogenic intrusion, and has been associated with increased risk of BV and STI [253–255]. The driving mechanism of this association remains contentious, with multiple explanations available; (i) pubic hair removers may be more likely to engage in sexually risky behaviours explaining the STI association, (ii) injuries from pubic hair removal may facilitate the permeation of STIs beyond the skin [254]. Hence, the methods of removal may incur different risks, (iii) alteration in grooming patterns may result in dysbiosis and enable STI acquisition [256].

Of additional concern is the global increase in genital cosmetic procedures such as labiaplasty [257], which surgically alter the labia for aesthetic sake [258–260]. Australian physicians have reported dramatic increases in labiaplasty requests, with blame being attributed to pornography and social media [260–262]. Given that aesthetic improvement and sexual function are key motivating factors in seeking cosmetic genital procedures [263], there is little known evaluation of these procedures on VMB composition. Some studies advocate that particular labial formation may contribute to recurrent urinary tract infections (UTIs), offering labiaplasty as a potential solution [85, 264]. However, this remains hotly contested [86], and it should be noted that this approach remains inaccessible for many AFAB individuals.

#### Antibiotic use

Antibiotic use has been proven to profoundly shift VMB composition [265, 266], yet is also driven by social elements; jurisdictional and socio-economic factors dictate access to antibiotics, as well as treatment modalities. Antibiotic overuse is known to cause increased AMR, primarily through the proliferation and transmission of AMR genes (ARGs), and thus deserves thorough investigation when considering STI transmission, emergence and evolution within vaginal micro-environments. Biologically, this may occur through vertical transmission from parent to daughter cells, or between microbes via several horizontal gene transfer (HGT) mechanisms [267]. Flora in the VMB may also carry, develop, and transfer ARGs, with the vaginal ‘resistome’ acting as an under-researched ARG reservoir which is especially relevant to the context of STIs [232, 268]. Given this in the context of biosocial drivers, it becomes clear that some subgroups may be disproportionately impacted due to confounding elements of biology and social disparity. Disruption of the VMB’s delicate balance by antibiotic use can lead to BV, or select for *Candida* spp. overgrowth (clinically known as ‘thrush’) [269]. Given that both BV and thrush may arise opportunistically from dysbiosis and often accompany pathogenic intrusion [270], it could be argued that dysbiosis acts as an indicator which provides some benefit to the host through symptomatically signalling a need for treatment. This argument is strengthened by the understanding that STIs are more likely to be asymptomatic in women [271–274], which might indicate a protective nature of dysbiosis symptoms in vaginal microenvironments. Altogether, this underscores the need to understand both the VMB balance and polymicrobial mechanisms, which may contribute to AMR-STI acquisition, and how different subgroups with varying physiology and sexual behaviours experience VMB fluctuation.

Antibiotic use as a driver of AMR-STI evolution is a truly biosocial problem and warrants extensive investigation. Whilst an individual may dictate their antibiotic consumption, their autonomy is somewhat limited given that antibiotic prescription is either regulated by medical authority, or available without oversight due to jurisdictional policy. Policies are an important consideration as population level drivers of ARG emergence and proliferation [275], and can act as selective pressures for specific ARGs. This is because they influence the likelihood of exposure to AMR-STIs, the relative impact an individual might have on their VMB composition over time, and an individual’s capacity to successfully treat AMR-STIs. For instance, a ‘test before treatment’ model is the gold standard antibiotic stewardship practise. However, in rural Australian and other LMIC settings, SRH care can be quite inaccessible with patients unlikely to seek follow up, hence physicians may choose to treat based on syndromic presentation alone, acting as a selective pressure through cumulative appointments in such areas. If AMR-STIs are in circulation in such areas and treatment guidelines are not sufficiently adjusted, then the likelihood that the first line treatment might fail for any given individual increases, which circularly reinforces the selection for the AMR-STI in question. Moreover, policy can influence individuals within the same jurisdiction differently due to inequities in underpinning social contracts. In our previous Doxy-PEP example, we highlighted that Doxy-PEP is available to AMAB but not AFAB GBMSM despite sharing the same sexual network and therefore AMR-STI acquisition risk. In the current context of increasing AMR-STIs known to circulate amongst GBMSM, this drastically heightens the relative risk experienced by AFAB GBMSM through several mechanisms: (i) it applies a selective pressure to GBMSM sexual networks for the emergence and dominance of tetracycline-resistant ARGs, (ii) the marketing of Doxy-PEP as a ‘morning after pill for STIs’ encourages condom complacency in the GBMSM community thereby increasing transmission risk, and (iii) it normalises a different standard of care for AFAB and AMAB GBMSM, imbuing inequity at a policy level which may create distrust and opportunities for the negative impacts of mis- and dis-information efforts. This is a perfect example of how intersecting social practises can drive biological phenomena, and a clear demand for improved equity in addressing the burgeoning problem of AMR-STIs.

### An ecological niche for AMR development

**Fig. 3:**
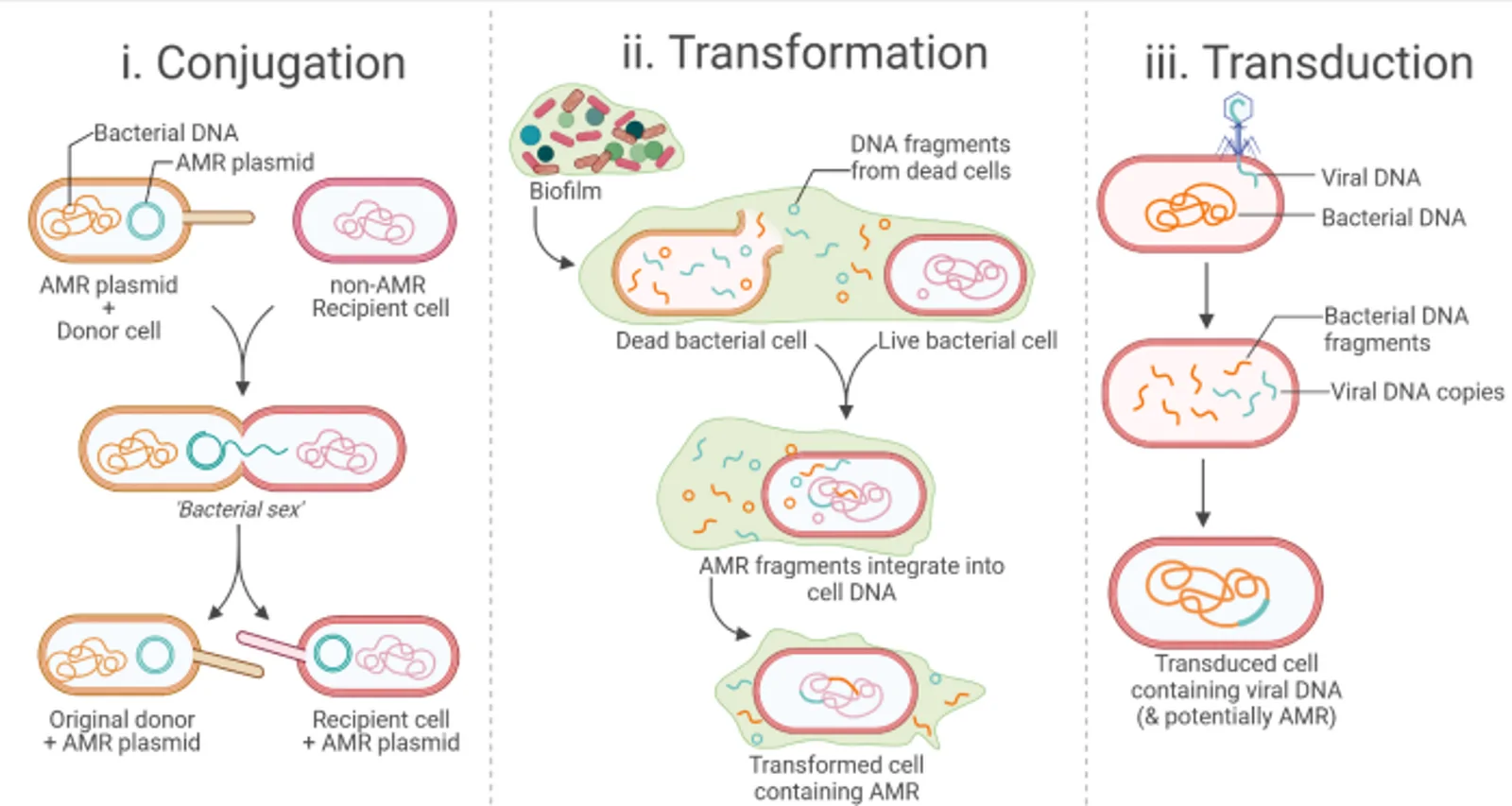
A summary explanation of horizontal gene transfer (HGT) facilitates AMR. HGT is well-understood to occur through three key mechanisms: conjugation, transformation, and transduction. (i) Conjugation describes a scenario commonly referred to as ‘bacterial sex’ wherein conjugative cellular machinery from one cell initiates genetic information transfer with another cell. During conjugation, bacterial cells are both actively engaging in HGT, enabling the transfer of ARGs through plasmid exchange from the donor cell to the recipient cell. (ii) Transformation occurs when living bacterial cells ‘pick up’ DNA fragments from their surrounding environment, usually from lysed bacteria. These fragments are then integrated into the cellular DNA or retained as plasmids, enabling the cell to express its new genes (such as ARGs). Biofilms are contextually relevant in the vaginal microenvironment, are known to increase HGT through increased cell proximity, and likely increase transformation efficiency through the EPS acting as a medium for fragmented cell retention. (iii) Transduction, also known as ‘phage pollination’ occurs when bacteria specific viruses infect bacterial cells unsuccessfully, enabling bacterial cellular immune systems to retain portions of phage DNA, which may also contain ARGs, especially if phage had previously encountered different bacterial cells. AMR – antimicrobial resistance; ARG – antimicrobial resistance gene; HGT – horizontal gene transfer; EPS - extracellular polymeric substance. Created in BioRender, adapted from [295]

Current STI treatments rely on antimicrobial drugs to kill or inhibit invasive pathogens. Antimicrobials are classed according to their predominant mechanism of action, with multiple derivatives of each class available for use as drug treatments. For example, doxycycline is a tetracycline derivative. ‘Tetracyclines’ are an antimicrobial class which operate by binding to cellular protein synthesis machinery, inhibiting bacterial replication and ultimately starving pathogens. Pathogens may avoid starvation through possession of tetracycline-resistant ARGs. As such, treatment regimens such as Doxy-PEP act as selective pressures for microbes with tetracycline-resistance-conferring ARGs. These ARGs may range from encoding efflux pumps which eject tetracyclines, to biofilm formation genes which reduce pathogen exposure to tetracycline action. Most importantly, microbes can exchange genetic information with one another through HGT [Figure 3], and ARGs are numerous, spanning all known classes of antimicrobials.

Presently, vaginal microenvironments serve as niches primed for ARG emergence due in large part to compounding social issues limiting progress in understanding VMB ecology and resistomes. One recent landmark study [232] investigated co-abundance of ARGs within the VMB, reporting inappropriate antibiotic use and low compliance were positively associated with increased ARG prevalence, whilst heightened AMR awareness and past compliance were conversely negatively associated with select ARGs [232]. This supports existing evidence that stewardship practices are crucial in limiting ARG emergence and widespread proliferation [276, 277], although expansion of this work beyond the relatively small sample size and ethnicity is needed to confirm mechanistic relationships. The protective mechanism of Lb spp. was further confirmed as Lb was negatively associated with all ARGs, although select Lb demonstrated positive associations with specific ARGs [232], reaffirming the capacity for commensal bacteria to acquire and harbour ARGs [278]. Taken together, this underscores the need for antimicrobial stewardship practises and well-considered policies in clinical settings, as complacency and antimicrobial overuse can clearly promote ARG emergence and retention in VMB commensals. The aforementioned study also found significant positive associations between *Candida* spp. presence and total ARG score [232], reaffirming that antibiotics promote thrush through ecological VMB disruption [269, 279], as well as ARGs. Recurrent candidiasis treatment may also explain this phenomenon if *Candida* spp. presence acts as a proxy for long term antimicrobial use and complacency. This is plausible given that azole treatments are typically available over the counter and are known to increase antifungal resistance [280], yet effects on endogenous bacteria have not been explicitly studied despite evidence for cross-kingdom HGT [281] and antimicrobial properties of novel azole derivatives [282]. Nonetheless, the relationship between thrush and BV appears complex and potentially mutually exclusive [269]. Most importantly, the recent landmark study found that alpha-diversity increased alongside ARGs per sample, with heightened ARG burden associated with increases in BV-associated taxa [232]. In particular, *Anaerococcus* spp., *Prevotella* spp. and *Dialister* spp. were implicated in promoting macrolide and tetracycline resistance, although the authors advocate that HGT and commensal transfer via the gut-vagina axis are likely involved [232, 283].

This study also reported several findings linking behaviours with ARG presence. Firstly, they found that oral contraceptive pills were negatively associated with *tet(M)* [232], indicating that their influence on VMB health may extend beyond their previously reported selection for Lb [170, 284]. Several studies have reported correlations between heightened body mass index (BMI) and increased ARG presence [232, 285–287]. However, there is a growing understanding that BMI is a poor measurement of health given its inconsistency across race, age, sex, ethnicity, and exercise habits [288–290], rendering this finding relatively unhelpful.

Evidently there is a substantial knowledge gap regarding AMR-STIs, the protective nature of VMB ecology and its direct relevance to SRH, as well as the quality of life and sexual function, especially in SGM and AFAB people outside of ‘reproductive age’. Furthermore, recurrent vulvovaginal infections, UTIs, and STIs appear to have beyond additive effects in terms of acquisition and host colonisation [291–294], although the ecology of the vaginal microenvironments remain little understood. To begin addressing this, we provide discourse regarding ecological interactions of some relevant STI pathogens within the VMB, their AMR status, and a quick-reference summary table [see Table 1] as a jumping-off point for future research.

**Table 1:** Pathogens in the VMB

Condition	Microbe	Type	Doubling time; morphology	Treatment	Mechanism of growth and survival in the VMB	Treatability and AMR	Ref
Candidiasis	*Candida albicans*,* Candida glabrata*	Fungal opportunistic pathogen	~ 2 h; polymorphic yeast	Clotrimazole; boric acid; nystatin	Forms complex biofilms containing microbial symbionts; multiple virulence factors	MDR strains increasingly emergent; biofilms can be resistant to immune responses and drug treatments; evidence for HGT-led AMR	[354–357]
Gonorrhoea	*Neisseria gonorrhoeae*	Bacterial STI	~ 1 h; Gram negative diplococcus	Ceftriaxone + Azithromycin; Ciprofloxacin	Evades host immune response. Evidence for upregulated expression of biofilm forming genes in vaginal microenvironments	MDR and XDR strain prevalence increasing; resistance to all antibiotic classes detected; extensive evidence for HGT and HGT-led AMR	[336, 358–364]
Chlamydia	*Chlamydia trachomatis*	Bacterial STI	~ 1.8 h; Gram negative obligate intracellular parasite	Doxycycline; Azithromycin	Hijacks cellular machinery to self-replicate or persist	Remains highly treatable; low AMR emergence; evidence for possibility of AMR-conferring mutations and HGT	[350, 365–369]
Syphilis	*Treponema pallidum*	Bacterial STI	~ 30 h; Gram negative, motile, spirochete	Benzylpenicillin	Evades host immune response and invades host cells	Remains highly treatable; increasing emergence of macrolide resistance	[348, 370–374]
*M. genitalium* infection	*Mycoplasma genitalium*	Bacterial STI	~ 12 h; facultative intracellular pathogen (lacks cell wall)	Doxycycline, Azithromycin/Moxifloxacin	Adheres to and invades host cells using specialised cellular machinery	Intrinsically resistant to cell-wall-targeting antibiotics; AMR to macrolides, fluoroquinolones. Evidence for novel HGT mechanism	[343, 345, 346, 375, 376]
Trichomoniasis	*Trichomonas vaginalis*	Protozoal STI	~ 6 h; flagellated protozoan	Metronidazole	Adheres to host cells, evades immune system, and seems to interact with vaginal microflora	Increasingly resistant since 1990. Significant evidence for HGT between bacteria and *T. vaginalis*	[377–381]
Bacterial vaginosis (dysbiosis, UTI, or STI)	*Escherichia coli*	Bacterial pathogen	~ 20 min; Gram negative rod-shaped	Nitrofurantoin; amoxicillin-clavulanic acid; gentamicin	Capable of glycolysis; forms intracellular reservoirs in place of Lb within eVMB cells	MDR increasingly emergent (beta-lactams, macrolides, tetracycline, aminoglycosides, fluoroquinolones); evidence for HGT	[382–387]
	*G. vaginalis* *A. vaginae* *Mobiluncus spp* *Prevotella spp*	Bacterial commensals/opportunistic pathogens	~ 1–24 h; primarily Gram negative or Gram variable rod-shapes	Metronidazole; Clindamycin	Evidence indicates BV may be sexually transmitted. Forms polymicrobial biofilms.	Polymicrobial biofilms facilitate HGT. Gv exhibits HGT capacity & may retain AMR genes.	[320, 326, 339, 388–391]

MDR = multidrug resistant, XDR = extensively drug resistant, h = hours, eVMB = oestrogen dominant vaginal microbiome

#### Lb in the VMBs

As established, Lb-dominance is supported by glycogen production in the epithelial cells of the eVMB, with Lb-c demonstrably protective of reproductive health. Lb populations are typically rich in intraspecies diversity [296], which may provide critical community stability through buffering against perturbations [26], and are mostly negatively associated with ARGs [232].

It is useful to discuss Lb-c and Lb-i due to their respective most and least protective natures in the VMB. Lb-c is a gram positive, facultative anaerobe that ferments glycogen to produce lactic acid, thereby contributing to acidic pH observed in vaginal microenvironments [297]. Interestingly, Lb-c, along with some rarer Lb, produce both D-lactic acid and hydrogen peroxide, whereas Lb-i does not, offering potential explanations of their different protective efficacies [298]. Finally, Lb-c dominance in the VMB operates as a “pathogen resistance” mechanism in and of itself, which appears to preclude the proliferation of alternative microbes [297, 299]. Recent landmark studies found evidence that a co-abundance group comprised of particular Lb spp. (Lb-c, *L. jenesenii*,* L. gasseri*) was negatively associated with ARG prevalence, lending in situ credibility to the concept of ‘protective’ vaginal CSTs [232, 300].

However, not all Lb provide equivalent host benefits, and a growing body of evidence suggests that even Lb commensals may acquire and harbor ARGs to facilitate adaptation to their dynamic microenvironment. A recent landmark study identified positive associations between *L. gasseri* and *erm(A)*, Lb-i and *tet(Q)*, and between unclassified Lb members and *qnrA*, supporting the notion that Lb may acquire ARGs from neighbouring bacteria through HGT [232]. Several factors may exacerbate or precipitate this phenomenon, including both widespread personal antibiotic overuse and commercial exploitation of antibiotics in the food chain where Lb act as starter cultures [232, 301–303]. Long considered a ‘transitional’ species enabling BV through poor protection, Lb-i has been associated with youth, multiple sexual partners, and unprotected sex [232, 304]. Several studies have corroborated that Lb-i is essentially health-neutral in the VMB [232, 305], and is commonly recovered from both healthy and BV-exhibiting VMBs. It is also perhaps the most common vaginal bacteria [298, 306], has the smallest Lb genome, limited metabolic capabilities, and expresses a cytolysin (enabling resource-liberation from host cells), which may explain the instability of Lb-i dominant VMBs [307–309]. Lb-i colonises during post-VMB-disturbance, explaining its lesser protection and therefore heightened BV, STI, and adverse health outcomes, earning the title of opportunistic pathogen despite its otherwise symbiont nature [310]. Genomic investigation has indicated certain genes are related to glycogen metabolism across a variety of microbes [103, 296].

Further research is needed to elucidate the role of Lb as a reservoir for ARGs in the context of STIs, although several studies have begun investigating the capacity of Lb to transfer ARGs to pathogenic strains [301, 302]. This is particularly relevant given tetracycline and macrolide resistance are used to treat the majority of STIs, and Ng, which has developed resistances to all known classes of antimicrobials and may harbour the *tet(M)* plasmid, conferring it with high-level tetracycline resistance [311, 312].

#### Polymicrobial STIs: BV and thrush

As previously mentioned, both BV and thrush (also known as vulvovaginal candidiasis or VVC henceforth) arise from VMB dysbioses and can be considered polymicrobial infections that are either sexually transmitted or caused by sex-driven VMB disturbance. Additionally, although their causative agents differ, in both cases they act as either commensals or opportunistic pathogens. Both have been associated with poor reproductive health outcomes and chronic recurrence [269, 313–315], and each produce biofilms which are implicated in increased risk of STI acquisition [316]. They are also each typically diagnosed based on syndromic clinical features, which presents a common complication of STI care for individuals with AFAB physiology. We discuss them summarily here, considering their similar roles within the VMB ecosystem.

Although the causative agent of VVC is known to be *C. albicans* (Ca), the role of the VMB remains contentious due to consistently conflicting findings regarding Lb colonisation and vaginal acidification, especially since Lb colonisation and pH are usually normal (< 4.5) during VVC infections, and Ca is commensal [317]. Clinical diagnosis of VVC is typically made by signs and symptoms of inflammation, pain, itching, and thick curd-like vaginal discharge, followed by a wet-mount slide indicating yeast presence [318]. Conversely, the complex polymicrobial nature of BV and a persistent inability to determine its specific causative agent have resulted in a diagnostic situation wherein several approaches have been offered by clinical, microbiological and molecular fields [319]. Although Gv is strongly suspected to play a causative role in BV, its common presence in healthy VMB phenotypes has been relatively controversial, with several BV-associated bacteria identified [27]. Ultimately, whilst BV was recently confirmed to be sexually transmissible through successful treatment of male partners [320], VVC is not classified as such.

From a microbial ecology perspective, virulence factors and biofilm formation are key considerations, and Ca and Gv each possess both. In BV infections, vaginolysin (a Gv virulence factor) facilitates the breakdown of vaginal epithelial cells [27], whereas in VVC infections, candidalysin appears to perform a similar, albeit more complex function [321]. VVC infections also benefit from several hydrolases, facilitating invasion and nutrient acquisition, and genes regulating expression are important in Ca biofilm development [317, 322, 323]. Biofilms are common in polymicrobial infections and can be defined as structures comprised of microbes and their self-secreted extracellular polymeric substance (EPS), which adhere to surfaces, protecting their resident microbes [324]. Biofilms are crucial to BV pathogenesis, and biofilm disruptors are central to emergent treatment approaches [325]. In BV infections, Gv is considered the primary coloniser responsible for the establishment of the EPS scaffold, with its biofilm-associated *sialidase A* gene producing sialidase, to aid in the degradation of vaginal mucus barriers [27, 326]. Known to demonstrate heightened tolerance to both lactic acid and hydrogen peroxide, Gv-biofilms are key proponents of vaginal health produced by Lb dominant VMBs [26, 327, 328]. Notably, Gv-biofilms demonstrate reduced metabolic capability and down-regulated vaginolysin [27], which could have implications regarding their persistence. Similarly, Ca biofilm pathogenesis involves adherence to vaginal epithelial cells, invasion, and the subsequent formation of biofilms [317]. Ultimately biofilms are relevant in AMR-STI emergence as they both facilitate HGT through increased microbial proximity and act as a barrier to antimicrobial treatments.

#### STIs of concern for AMR and HGT

Several AMR-STIs are of concern given their capacity and likelihood to engage in AMR through HGT. For instance, the highly mutable Ng (causative of gonorrhoea) is a pressing global health concern and has evolved widespread ARGs to all known classes of antimicrobials [329]. A 2020 global study estimated Ng incidence at 82.4 million adult cases [330], with substantial increases reported since [331]. Crucially, although Ng disproportionately affects specific subpopulations including SGM and socioeconomically disadvantaged people [332], biological investigation as to why remains limited. Gonococcal infections are widely considered more likely to be asymptomatic in women, although this belief likely stems from differences in AFAB physiology combined with comparatively poorer clinical diagnosis [333]. In terms of VMB ecology, Lb-dominance has been demonstrated to reduce Ng infection risk, primarily through adherence inhibition [317, 334, 335], solidifying its role as a protective mechanism. Transcriptomic investigation (which uses RNA to interrogate gene expression) has confirmed differences in Ng gene expression between genders, highlighting selective pressures of the microenvironment [336]. Future mechanistic studies should carefully consider the impact of VMB context in conjunction with sexual network and transcriptomic trends to accurately clarify nuance. Its cellular machinery makes Ng prolifically capable of ARG development and dissemination to other microbes in the VMB, whilst its natural competence increases its propensity for HGT [268, 333]. It is also capable of forming polymicrobial biofilms, further increasing its propensity for ARG acquisition [337–339]. This means that Ng can not only ‘learn’ and replicate ARG strategies from other microbes, but may also act to disseminate them, meaning we must turn our attention to receptive microbes which would seek ARGs as an evolutionary benefit. This primarily includes opportunistic gut or skin bacteria that cause urinary tract infections, such as *Escherichia coli*, *Streptococcus* spp., *Klebsiella pneumoniae*,* Enterobacter* spp., *Pseudomonas aeruginosa*, *Enterococcus* spp., and methicillin-resistant *Staphylococcus aureus* (MRSA) [268, 340]. Arguably, this should also be expanded to include *Shigella* spp., which are considered an STI of concern in GBMSM [341, 342], as they also presents a risk for SGM individuals engaging in vaginal penetration with GBMSM. This may include AMAB individuals with neovaginal physiology, or AFAB GBMSM with vaginal physiology and tVMB or eVMB balances to consider. Transfer of plasmids between each of these pathogens is probable, with *E. coli* acting as the model organism for most HGT studies. However, exact interactions within VMB ecology and physiology remains relatively underexplored. The capacity of microbes to engage in methods of HGT depend largely on their cellular machinery (Fig. 3). Hence, they may act as either donors, recipients, or both.

For instance, *Mycoplasma genitalium* (Mgen) is the smallest of the STIs and has undergone such significant genomic reduction that it is metabolically reliant on host cells, functioning similarly to an intracellular parasite [343]. As a result, it now benefits from intrinsic resistance to drugs targeting irrelevant cellular machinery, leaving just tetracyclines, fluoroquinolones, and macrolides, which operate as the mainstay antibiotic despite increasing reports of resistance [343, 344]. Whilst Mgen is unlikely to receive genes via HGT due to its small size and lack of recipient machinery, it may instead act as a donor through mycoplasma chromosomal transfer, increasing the AMR capacity of recipients. Close relatives of Mgen can leverage conjugative machinery to achieve this [345], and preliminary evidence suggests that through regulation by the RecA protein, Mgen also has this capacity [346]. This concern is supported by the consistent observation of relatively high rates of co-infection with both Mgen and other STI pathogens [343]. This of course could have wide-reaching implications for treatment failure if resistance continues to increase, especially given the complex nature of VMB ecology. Considering this in the context of the proven capacity of Lb to act as an ARG reservoir, future studies should further investigate HGT mechanisms between pathogens and commensals, as well as the retention capacity following treatment of various VMB compositions.

#### STIs unlikely to engage in HGT

Finally, some STIs are unlikely to engage in HGT due to a lack of evolutionary fitness benefit or necessary cellular machinery. Since transformation-competent microbes can use lysed DNA fragments, non-HGT STIs may still act as passive donors, although their risk of developing and disseminating ARGs is arguably reduced comparative to their more active counterparts. Syphilis and chlamydia are two such STIs, which are more likely to develop AMR through increased exposure to antimicrobials, as opposed to HGT, and each possess different metabolic and pathogenic pathways. Syphilis, for instance, is caused by *Treponema pallidum*, a spirochete with a lengthy doubling time, meaning that infections (and therefore mutations) take a relatively long time to develop, with limited evidence for any utility or evolutionary benefit of HGT [347, 348]. Although syphilitic infections remain treatable with penicillin to date, widespread macrolide resistance has been reported in syphilis, aligning with historical overuse [348], demonstrating its capacity for AMR development. *Chlamydiae trachomatis*, the causative agent of chlamydia, is similarly affected by macrolide resistance due to genetic mutations [349]. Although it also remains treatable with mainstay antibiotics, its close relatives have demonstrated capacity to AMR development in response to heightened antimicrobial exposure [350]. It could be argued that despite concerning increases in syphilis and chlamydia, their continued treatability and the necessity of retaining antimicrobial stewardship practises to avoid AMR emergence renders interventions like Doxy-PEP irresponsible. Syphilitic and chlamydial infections both cause disproportionately severe SRH impacts to AFAB individuals with vaginal physiology [351–353]. This only underscores the concern and highlights the risk-increase faced by AFAB GBMSM due to systematically ineffectual policy. Future research into sexual networks and relevant physiological selective pressures may yield insight, which may lead to improved treatment policies.

### Summary of outstanding research questions

Despite a drastic improvement in VMB research efforts in the last decade, several key areas remain to be investigated. To address this, integrated public health strategies combining (i) antibiotic stewardship, (ii) lifestyle interventions, and (iii) sexual network risks would provide substantial, population-level benefit. Personalised therapeutic approaches tailored to VMB composition, resident resistome, and physiology, represent not only a necessary paradigm shift, but also potentially the most promising avenue for the development of novel treatment strategies. Historically, each VMB composition has been approached in the context of its vaginal microenvironment at different rates, resulting in various knowledge gaps.

Although eVMB sequencing efforts have yielded DNA detection facilitating insight into composition, this research is still limited. However, such research would benefit from (i) differentiation of live and dead bacteria to understand relational roles, (ii) meta-omic investigations into the roles of fungi, parasites and viruses within a largely bacterial context, (iii) linked longitudinal vulvar microbiome investigations, (iv) investigation of sexual practises from an anatomical-site perspective, and (v) linkage with investigation of ARG presence and expression [1, 2]. Furthermore, studies should be adequately powered per population group, with an ability to consider ethnicity, race, and SGM [1]. Lastly, menstrual phases and pregnancy status have proven unequivocally important to the eVMB [1]. Hence, studies should be structured to reflect this, or to reduce interference of these variables in trend analysis. In addition to suggestions for the eVMB, the tVMB and post-menopausal VMB warrant significant further study, with a focus on linking symptoms with VMB composition, hormonal influence, and sexual practises. Longitudinal studies of the transition from eVMB to tVMB composition following testosterone therapy would be of great benefit to AFAB SGM [1] and may provide direct treatment approaches for dysbiosis to this group. Additionally, the effects of topical oestrogen therapies on tVMBs and post-menopausal VMB composition is a key first step in providing meaningful SRH care to AFAB SGM and post-menopausal women. Finally, neoVMB research is in its infancy. Hence, further research is critical in providing SRH care to AMAB SGM. Tissue source and placement during neovaginal construction is of critical importance to neoVMB composition, and any future research should frame this as a key component, ensuring sufficient power to address tissue origin [1]. Linkage of tissue source, symptoms, VMB composition, and sexual networks and practises is an important next step in this line of research to determine a classification framework for healthy versus pathological conditions.

## Conclusions

The human VMB forms an ecological niche home to a complex ecosystem of microorganisms. Its composition is demonstrably diverse between individuals, with variations observed across racially and ethnically diverse groups, and intrapersonal fluctuations linked to a plethora of factors both within and outside of personal control. Sex, gender, and sexuality are inextricably linked to both VMB composition and STI acquisition, yet practices in medical research have failed to account for their evidently complex interplay. This has led to a situation wherein individuals with vaginal physiology suffer inequitable SRH care and are disproportionately impacted by STI-associated sequalae. Social context not only shapes SRH care, but can also act as a driver of biological phenomena, including VMB ecology and AMR-STI acquisition. This is especially relevant in the intersecting contexts of AMR-STIs and SGM, which culminate in a pressing issue for the future of SRH care on a global scale. We also highlight the several factors which culminate in the VMB serving as an understudied reservoir for AMR. Finally, we highlight several important knowledge gaps to benefit from further research. We hope that this review serves to drive future research efforts, highlighting the social context of vaginal microbiomes, and their potential influence on AMR-STI development.

## Data Availability

No datasets were generated or analysed during the current study.

## Abbreviations

AFAB: Assigned female at birth
AMAB: Assigned male at birth
AMR: Antimicrobial resistance
AMR-STI: Sexually transmitted infection demonstrating antimicrobial resistance
ARG: Antimicrobial resistance gene
BMI: Body mass index
Ca: Candida albicans
Cis: Cis-gendered, meaning that an individual’s gender identity is concordant with their gender assigned at birth
Cis-het: Cisgendered heterosexual
Doxy-PEP: Doxycycline post exposure prophylaxis
EPS: Extracellular polymeric substance (essentially the ‘glue’ of biofilms)
eVMB: Oestrogen-dominant vaginal microbiome
GBMSM: Gay or bisexual men who have sex with men
Gv: Gardnerella vaginalis
HIV/AIDS: Human Immunodeficiency Virus / Acquired Immunodeficiency Syndrome
HGT: Horizontal gene transfer
HRT: Hormone replacement therapy
Lb: Lactobacillus
Lb-c: Lactobacillus crispatus
Lb-i: Lactobacillus iners
LGBTQIA+: Lesbian, gay, bisexual, transgendered, queer/questioning, intersex, asexual and other identities pertinent to the broader queer community
Micro-ecology: Shortened from ‘microbial ecology’, this refers to the interactive microbiological ecosystem of microbes which involves environmental factors and co-inhabitants alike
neoVMB: Neovaginal microbiome (notably distinct through surgical neovaginal construction)
Ng: Neisseria gonorrhoeae
SGM: Sexuality or gender diverse minorities (these are essentially subsets of the LGBTQIA+ community)
spp.: Species
STI: Sexually transmitted infection
Trans: Trans-gendered, meaning that an individual’s gender identity is not concordant with their gender assigned at birth
tVMB: Testosterone-dominant vaginal microbiome
UTIs: Urinary tract infections
VMB: Vvaginal microbiome
VVC: Vulvovaginal candidiasis
WSW: Women-who-have-sex-with-women
